# The Type VI Secretion System: A Dynamic System for Bacterial Communication?

**DOI:** 10.3389/fmicb.2017.01454

**Published:** 2017-07-28

**Authors:** Mathias Gallique, Mathilde Bouteiller, Annabelle Merieau

**Affiliations:** Laboratoire de Microbiologie Signaux et Microenvironnement EA 4312, l’Institut Universitaire de Technologie d’Evreux (IUT), Université de Rouen, Normandy University Evreux, France

**Keywords:** type six secretion system, quorum sensing, communication, social behavior, membrane perturbation

## Abstract

Numerous studies in Gram-negative bacteria have focused on the Type VI Secretion Systems (T6SSs), Quorum Sensing (QS), and social behavior, such as in biofilms. These interconnected mechanisms are important for bacterial survival; T6SSs allow bacteria to battle other cells, QS is devoted to the perception of bacterial cell density, and biofilm formation is essentially controlled by QS. Here, we review data concerning T6SS dynamics and T6SS–QS cross-talk that suggest the existence of inter-bacterial communication via T6SSs.

## Introduction

Bacteria are perpetually at war against multiple competitors and thus require weapons to conquer new territory or persist in an ecological niche. Among the mechanisms that aid in the struggle against other bacterial species are the Type VI Secretion Systems (T6SSs) (Hood et al., 2010). The T6SSs of Gram-negative bacteria are effector translocation apparatuses, resembling an inverted bacteriophage-puncturing device, composed of at least 13 proteins called core-components (TssA-M, for Type six secretion) (Boyer et al., 2009; Silverman et al., 2012). Auxiliary components can be associated with these conserved proteins (Tag, for Type six secretion associated genes) (Shalom et al., 2007). T6SSs participate in a broad variety of functions, including virulence and antibacterial activity (Pukatzki et al., 2006, 2007; Ma et al., 2009; Coulthurst, 2013; Sana et al., 2016). T6SSs also participate in metal ion uptake, such as that of iron, manganese, and zinc (Wang et al., 2015; Chen et al., 2016; Lin et al., 2017; Si et al., 2017), conferring an advantage during bacteria–bacteria competition. In this review, we provide an overview of the data on T6SS assembly and emphasize connections between T6SSs and bacterial communication.

## T6SS Dynamics

### The Global Scenario

**FIGURE 1:**
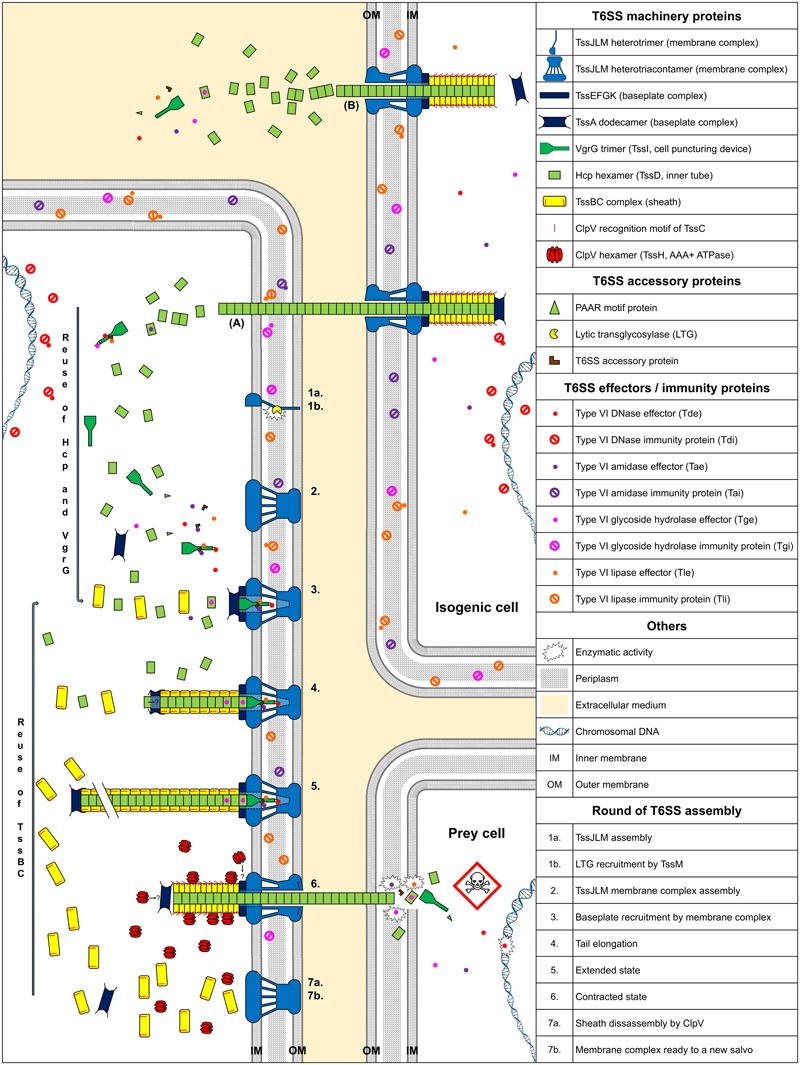
Model of T6SS assembly and Hcp/VgrG/effector translocation and recycling. The membrane complex is anchored to the cell envelope with the help of a lytic transglycosylase (1a, 1b, and 2). While effectors are loaded onto their respective Hcp, VgrG, or PAAR proteins, the baseplate platform is positioned (3), aiding inner tube and sheath assembly (4 and 5). TssA interacts with the membrane complex, allowing contractile tail polymerization (3). Hcp or sheath components can be individually added during elongation (4 and 5). Following sheath contraction, Hcp, VgrG/PAAR, with their associated effectors, are pushed out and delivered into prey cells (6). ClpV disassembles the sheath and probably other T6SS components (7a) and the membrane complex may be used for a new assembly cycle using recycled components (7b). **(A)** Indicates exogenous Hcp and VgrG that can be recycled after isogenic T6SS aggression. **(B)** Indicates secretion of Hcp and VgrG into the culture medium. The question mark indicates a hypothesis.

Type VI Secretion System contractile nanomachines allow bacteria to inject toxins directly into prey cell membranes or cytoplasm. The machinery of the T6SS is assembled in an orderly manner. It starts with membrane complex formation, allowing baseplate positioning. The baseplate serves as a platform for contractile tail elongation. Contraction of the sheath propels effectors across membranes. Finally, the ATPase, TssH (ClpV), recycles the sheath and probably other T6SS components such as TssA, whereas the membrane-anchoring complex can be used to fire a new salvo (**Figure 1**).

### Membrane Complex Assembly

Type VI Secretion Systems are anchored to the cell envelope by a membrane core complex (Durand et al., 2015), which serves as a T6SS docking station and platform for baseplate assembly and prevents membrane cell damage during effector injection. The membrane core complex is a 1.7 MDa structure with a fivefold symmetry composed of 10 heterotrimeric complexes containing the three proteins TssJ, TssL, and TssM. Hierarchical biogenesis of this complex is initiated by the insertion of the lipoprotein TssJ in the outer membrane (Aschtgen et al., 2008; Zoued et al., 2014). TssJ then interacts with the large periplasmic domain of the inner membrane protein TssM (Felisberto-Rodrigues et al., 2011; Nguyen et al., 2015). The cytoplasmic domain of TssM interacts with the inner membrane protein TssL and the cytoplasmic domain of another TssM subunit, thus enabling oligomerization (Logger et al., 2016). Similarly, the cytoplasmic domain of TssL mediates self-polymerization (Durand et al., 2012; Zoued et al., 2016a). The TssM periplasmic domain recruits a lytic transglycosylase (LTG), which is required for local peptidoglycan layer degradation, necessary for proper assembly of the 1.7 MDa TssJLM complex (**Figure 1**, assembly steps 1 and 2) (Weber et al., 2016; Santin and Cascales, 2017). Associated proteins with a peptidoglycan-binding domain, such as TagL, which can bind to truncated TssL, can associate with the membrane complex. TagL corresponds in this case to an “ancestral TssL” domain (Aschtgen et al., 2010a,b).

### Baseplate Complex Positioning

The T6SS baseplate complex, composed of TssA, TssE, TssF, TssG, TssK, and Valine-glycine repeat protein G (VgrG or TssI) proteins (Brunet et al., 2015), is recruited by the membrane complex (Zoued et al., 2013). This structure serves as a platform for contractile sheath assembly and is essential for the correct assembly of the inner tube, comprised of hexameric rings of Hemolysin-coregulated protein (Hcp or TssD) (Brunet et al., 2015). TssA forms a dodecamer complex, which first binds to the membrane complex (Zoued et al., 2016b) (**Figure 1**, assembly step 3). Positioning of the baseplate complex may be initiated by recruitment of TssE, TssK, and VgrG via TssA (Planamente et al., 2016; Zoued et al., 2016b). The cytoplasmic domains of TssM and TssL, located at the base of the membrane complex, interact with the TssG/TssK and TssK/TssE baseplate subunits, respectively (Zoued et al., 2013, 2016a; Logger et al., 2016). The baseplate complex thus forms an interface between the membrane complex and the T6SS tail: both the Hcp and TssC sheath subunits interact with baseplate components (Brunet et al., 2015) (**Figure 1**, assembly step 4). After TssE recruitment, TssA likely properly attaches the sheath onto the baseplate and/or stabilizes the sheath structure (Planamente et al., 2016).

### Elongation of the Contractile Tail

The TssE baseplate component may initiate sheath assembly and anchors the sheath to the baseplate (Kudryashev et al., 2015). Hcp proteins assemble into hexameric rings, stacked head-to-tail, under the control of baseplate components (Brunet et al., 2014, 2015). The TssBC sheath component then wraps around the inner Hcp tube (Zoued et al., 2016b). Formation of the inner tube precedes TssBC assembly and is primordial for proper stacking of the subunits (Basler et al., 2012; Kapitein et al., 2013; Brunet et al., 2014). The Hcp tube has an inner diameter of ∼40 Å (Mougous et al., 2006), forming a lumen with a neutral surface, suggesting passive effector translocation into the Hcp tube (Ge et al., 2015). The diameter of the internal sheath, measuring approximately 100–110 Å (Bönemann et al., 2009), coincides with the ∼80–85 Å outer diameter of the Hcp hexamer (Cascales and Cambillau, 2012). The TssA dodecamer is located at the distal end of the tail in *Escherichia coli* (Zoued et al., 2016b), whereas TssA1 is a component of the baseplate/tail subcomplex in *Pseudomonas aeruginosa* (Planamente et al., 2016). The TssA complex appears to possess short, flexible arm-like extensions, which may grasp TssBC or Hcp and incorporate them, one by one, at the distal end of the contractile tail (Zoued et al., 2016b). Moreover, the diameter of the central channel of the ring-shaped TssA structure measures approximately 100 Å (Planamente et al., 2016), comparable to the dimension of the Hcp hexamers. Hcp components perhaps pass through the large central lumen of the TssA dodecamer (**Figure 1**, assembly step 4) and are added to the growing structure. Contrary to bacteriophages, the length of the T6SS tail does not appear to be controlled by a specialized protein (Zoued et al., 2014; Vettiger and Basler, 2016). The length of the T6SS tail can exceed 1 μm (Basler et al., 2012). It is possible that contact with the opposite cell membrane is the physical signal to stop tail elongation (**Figure 1**, assembly step 5).

### Contraction and Sheath Disassembly

Clemens et al. (2015) demonstrated that the sheath of *Francisella tularensis* has a quaternary structure with handedness opposite to that of the contracted sheath of T4 phage tails. The sheath contracts within a few milliseconds (Basler et al., 2012), propelling the Hcp-VgrG spike and effectors, punching either indiscriminately or in a focused manner into neighboring bacteria. The sheath contracts and becomes shorter and wider than in the extended state (Basler et al., 2012). Once the sheath is contracted, the ClpV recognition motif of TssC, which is buried in the elongated state, becomes accessible (Bönemann et al., 2009; Pietrosiuk et al., 2011; Basler and Mekalanos, 2012; Kapitein et al., 2013; Kube et al., 2014; Douzi et al., 2016), permitting TssBC recycling by the ATPase. Thus, TssB and TssC can be reused for a new round of sheath elongation (**Figure 1**, assembly steps 6 and 7). An alternative mode of sheath disassembly may involve the TagJ accessory protein (Forster et al., 2014). TagJ is structurally related to particular TssA C-terminal extensions (Planamente et al., 2016). In this case, TagJ interacts with TssB and recruits ClpV to the sheath (Forster et al., 2014). ClpV can also interact with TssA and may be involved in recycling TssA rings (Planamente et al., 2016).

### Effector Translocation

The puncturing device, consisting of the VgrG trimer, is located at the top of the inner tube and may be crucial for piercing the prey cell envelope. The VgrG trimer sometimes terminates with a Pro-Ala-Ala-Arg (PAAR)-repeat-containing protein, sharpening the tip (Shneider et al., 2013; Bondage et al., 2016).

Effectors transported by T6SS fall into two groups: “specialized” effectors and “cargo” effectors (Cianfanelli et al., 2016). Specialized effectors are extension domains of a structural component, whereas cargo effectors interact directly with VgrG, PAAR, or Hcp proteins (Silverman et al., 2013), with or without the help of accessory proteins (Alcoforado Diniz and Coulthurst, 2015; Liang et al., 2015; Unterweger et al., 2015). Four main classes of antibacterial effectors have been described, according to the target (**Figure 1**). Peptidoglycan targeting effectors are comprised of both Type six amidase effectors (Tae) and Type six glycoside hydrolase effectors (Tge) (Whitney et al., 2013). Type six lipase effectors (Tle) hydrolyse membrane phospholipids (Russell et al., 2013; Flaugnatti et al., 2016), whereas Type six DNase effectors (Tde) have nuclease activity (Ma et al., 2014). Some toxins do not belong to any of these four main classes. Pore-forming toxins, such as VasX from *Vibrio cholerae*, disrupt the inner membrane integrity of target cells (Miyata et al., 2013). Whitney and collaborators identified a NAD(P)^+^ glycohydrolase toxin in *P. aeruginosa* (Whitney et al., 2015). This toxin depletes cellular NAD(P)^+^ levels and induces bacteriostasis. The T6SS is not only an injection mechanism, it also enables the release of a proteinaceous metallophore into the extracellular medium and plays a role in the transport of Mn^2+^ under conditions of oxidative stress (Si et al., 2017) and iron uptake (Chen et al., 2016; Lin et al., 2017).

Bacteria that secrete antibacterial toxins also produce immunity proteins, which interact with toxic effectors, to allow self-protection and prevent the killing of sibling cells (called Tai, Tgi, Tli, and Tdi, corresponding to their effector family). Immunity proteins and effector targets are located within the same cellular compartment (Russell et al., 2013). Therefore, *tli* genes encoding outer membrane lipoproteins or periplasmic exposed lipoproteins, the Tle, should act in the periplasm (**Figure 1**).

Some other proteins secreted by the T6SS are involved in self-recognition, allowing communication between bacteria (Wenren et al., 2013; Cardarelli et al., 2015; Saak and Gibbs, 2016). In bacteria, secreted proteins are involved in many functions and are essential for bacterial fitness (Maffei et al., 2017). In some strains, the T6SS is activated in response of T6SS aggression by neighboring bacteria during cell–cell contact. Thus, the T6SS can modulate the fitness of other bacteria. In addition, T6SSs can be active, even in pure culture, and the presence of Hcp and VgrG in the culture medium is often used as evidence of a functional T6SS (Pukatzki et al., 2006, 2009). What purpose, however, does a functional T6SS serve in the absence of a competitor or prey?

## Is the T6SS Involved in Cell-To-Cell Signaling and Communication?

### Prelude

*Pseudomonas aeruginosa* is a widely-used model for T6SS studies. *P. aeruginosa* possesses three T6SS clusters comprised of TssA-M core component proteins. They are called H1-T6SS, H2-T6SS, and H3-T6SS. Among them, the H1-T6SS machinery is the most widely studied and is involved in antibacterial activity (Hood et al., 2010). The H2-T6SS and H3-T6SS are involved in virulence in eukaryotes (Lesic et al., 2009; Sana et al., 2012, 2015) but also display antibacterial activity by secreting trans-kingdom effectors, such as PldA and TplE via the H2-T6SS or PldB via the H3-T6SS machinery (Russell et al., 2013; Jiang et al., 2014, 2016).

### “Dueling” and “Tit-for-Tat”

**FIGURE 2:**
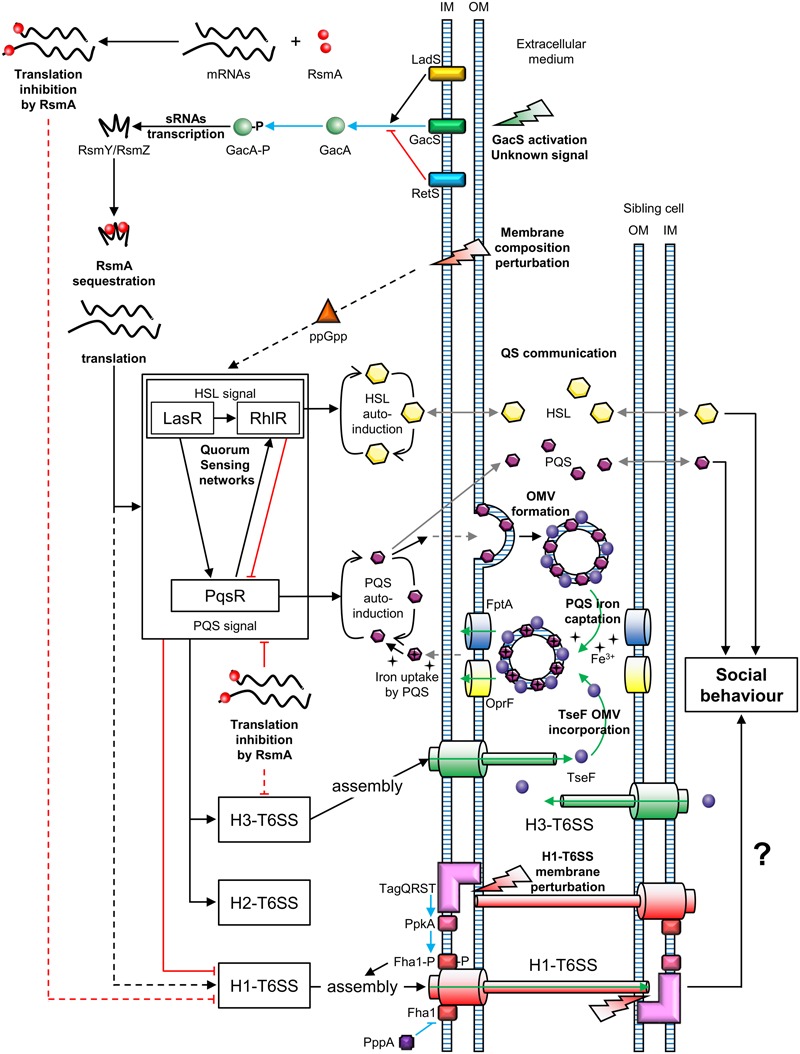
Connections between T6SSs and bacterial communication in *Pseudomonas aeruginosa*. OM, outer membrane; IM, inner membrane; OMV, outer membrane vesicle; P, phosphate; QS, quorum sensing. Green arrows represent molecule transport or uptake; black arrows indicate positive regulation; red blunt lines represent negative regulation; gray arrows represent diffusion; blue arrows indicate phosphorylation and blue blunt lines represent dephosphorylation; uncharacterized regulations are indicated by dotted lines. The question mark indicates a hypothesis.

Two types of T6SS behavior can be distinguished: that of defensive (targeted firing) and offensive cells (arbitrarily firing). *P. aeruginosa* can discern T6SS-mediated aggression of neighboring sister cells (Basler and Mekalanos, 2012). Similarly, *P. aeruginosa* can perceive T6SS attacks coming from *V. cholerae* or *Acinetobacter baylyi* cells (Basler et al., 2013). In both cases, *P. aeruginosa* is first assaulted by a nearby bacterium and then attacks, in turn, the aggressive cell. This mechanism is called “T6SS dueling” and is mediated by the H1-T6SS. Thus, *P. aeruginosa* only counterattacks in response to T6SS firing of *V. cholerae* or *A. baylyi*. In general, *P. aeruginosa* does not target T6SS-defective bacteria, suggesting that it is a defensive bacterium, in contrast to offensive *V. cholerae* and *A. baylyi* strains. However, *P. aeruginosa* strains affected in the hybrid sensor kinase RetS, can attack T6SS-defective bacteria in a H1-T6SS-dependent manner (Hachani et al., 2013, 2014). The perception of a T6SS attack involves the TagQRST threonine phosphorylation pathway, following envelope perturbation after T6SS-mediated perforation (Basler et al., 2013; Casabona et al., 2013; Ho et al., 2013) (**Figure 2**). Indeed, Polymyxin B, which alters cell membranes of Gram-negative bacteria, mediates activation of the T6SS, confirming that envelope perturbation triggers the T6SS counterattack (Ho et al., 2013). The TagQRST trans-membrane signaling cascade, essential for activation of the H1-T6SS of *P. aeruginosa*, is composed of four proteins. The ABC transporter complex TagST, anchored to the inner membrane, has ATPase activity (Casabona et al., 2013). In this complex, TagT is required for T6SS activation after cell membrane perturbation (Ho et al., 2013). TagQ, an outer membrane lipoprotein, is necessary for outer membrane localisation of TagR, which is required for protein kinase PpkA phosphorylation (Hsu et al., 2009). PpkA phosphorylates, in turn, Fha1 (which has a forkhead-associated domain), thus activating H1-T6SS assembly (**Figure 2**). The phosphatase PppA counteracts the role of PpkA by dephosphorylating Fha1 (Mougous et al., 2007). T6SS dueling appears to be an indirect means of communication, in which the occurrence of T6SS attacks may correlate with cell density. As the population increases, the likelihood of targeting sister cells also intensifies. In other words, as the population grows, the incidence of T6SS aggression rises. Thus, the perception of T6SS attacks provides an overall view of bacterial density and a form of social interaction (**Figure 2**).

### The GacS/GacA System and the Interplay between the T6SS and Quorum Sensing in *P. aeruginosa*

Two-Component Signal Transduction systems (TCSTs) are involved in external signal perception via a “sensor” and translate the signal via a “response regulator.” Thus, TCSTs play a key role in adaptive responses during environmental stress. GacS/GacA is a TCST in *P. aeruginosa* that perceives unknown signals and regulates numerous networks (Reimmann et al., 1997). The GacS/GacA cascade activates the transcription of the small RNAs (sRNAs) RsmZ and RsmY. RsmZ has a high affinity for the RNA-binding protein RsmA and can sequester it, permitting the translation of genes encoding H1-T6SS mRNAs. A *rsm*Z mutation results in downregulation of the transcription of genes encoding the H1-T6SS and H3-T6SS of *P. aeruginosa* (Brencic et al., 2009; Moscoso et al., 2011). The GacS/GacA system is under the control of two hybrid sensors, RetS and LadS. The hybrid sensor kinase RetS decreases RsmZ/RsmY transcription via the inhibition of GacS/GacA phospho-relay (Goodman et al., 2004). In contrast, LadS, enhances GacA phosphorylation via GacS (Chen et al., 2015). In summary, H1-T6SS is upregulated by the GacS/GacA/RsmZ regulatory pathway, which depends on the balance between RetS and LadS activation from external signals, unlike the H2-T6SS and H3-T6SS (**Figure 2**).

Quorum Sensing (QS) is a system that allows social synchronization, based on the perception of population density, according to signal molecule concentration. QS is crucial for collective adaptive responses (similar to a social behavior) and regulates both bacterial virulence and biofilm formation (Deng et al., 2011). *P. aeruginosa* has four QS networks consisting of three classes of diffusible auto-inducers (Lee and Zhang, 2015). The first class includes two types of Homoserine Lactones (HSLs): *N*-(3-oxododecanoyl)-Homoserine Lactones (odDHL, 3-oxo-C12-HSL) and *N*-butyrylhomoserine Lactones (BHL, C4-HSL) controlled by the Las and Rhl cascades, respectively (Schuster and Greenberg, 2007). *P. aeruginosa* also produces *Pseudomonas* Quinolone Signal (PQS), 2-heptyl-3-hydroxy-4-quinolone, of which the production is regulated by the PqsR cascade (also known as MvfR) (Cao et al., 2001). The last QS system consists of 2-(2-hydroxyphenyl)-thiazole-4-carbaldehyde, which is involved in the Integrated Quorum Sensing system (IQS) (Lee et al., 2013). The IQS can enhance PQS production depending on the *P. aeruginosa* strain (Lee et al., 2013; Sun et al., 2016). These QS networks are all interconnected and positively regulated by the Las cascade (Pessi et al., 2001; Lee et al., 2013). At the same time, RsmA is a negative post-transcriptional regulator of both 3-oxo-C12-HSL and C4-HSL production, affecting the Las*/*Rhl quorum sensing cascades (Pessi et al., 2001). Similarly, PqsR is post-transcriptionally repressed by RsmA (Kulkarni et al., 2014). However, the PQS system and Rhl cascade are upregulated via the Las pathway and PQS positively regulates the Rhl cascade (Rasamiravaka et al., 2015). In summary, the GacS/GacA system is a global activator of QS communication, because the action of the RNA-binding protein RsmA is jointly antagonized by RsmY and RsmZ (Kay et al., 2006) (**Figure 2**). At the same time, QS positively regulates H2-T6SS and H3-T6SS, whereas it suppresses H1-T6SS associated gene expression (Lesic et al., 2009).

A study published by Lin et al. (2017) revealed a link between the H3-T6SS and cell–cell signaling in *P. aeruginosa.* The cell–cell signaling compound PQS contributes to the formation of Outer Membrane Vesicles (OMVs) and associates with vesicle membranes. The PQS in OMVs can capture iron from the extracellular medium. In parallel, the protein TseF, secreted by the H3-T6SS, associates with OMVs containing PQS. The TseF from the OMVs then interacts with the Fe(III)-pyochelin receptor FptA and the porin OprF. This enables the delivery of PQS, associated with iron, into bacterial cells. Thus, the effector TseF delivered by the H3-T6SS is involved in the PQS pathway (**Figure 2**).

### T6SS and Social Behavior in *Proteus mirabilis*

*Proteus mirabilis* strains are able to recognize isogenic cells, coordinate multicellular swarming motility, and form macroscopic boundaries with non-sister cell swarms (Alteri et al., 2013). Macroscopic demarcation, called Dienes lines, can be observed between swarming *P. mirabilis* strains. Functional T6SSs are involved in this recognition phenomenon in the region of inter-strain contact. This visible boundary requires physical cell–cell interactions and is the result of T6SS recognition. Indeed, cells at the intersection between two swarming populations of *P. mirabilis* appear to kill each other using their T6SS effectors (Alteri et al., 2013). The T6SSs appear to assemble and fire deeply beyond the inter-strain boundary into the opposing swarming cells, thus enhancing T6SS effector injection (Alteri et al., 2013). Some Identification of self (Ids) proteins, involved in self-recognition and territorial behavior, are exported by the T6SS (Wenren et al., 2013). For example, IdsD mediates identity recognition between neighboring cells in a T6SS-dependant manner. IdsD interacts specifically with the cognate IdsE protein on the surface of recipient cells. The specific interaction between the two membrane-bound self-recognition proteins IdsD and IdsE drives social behavior (Cardarelli et al., 2015). These binding interactions contribute to the definition of strain identity and discrimination between self and neighboring non-self cells. The lack of binding between IdsD and IdsE correlates with the formation of the visible boundary. The authors speculate that IdsE itself contributes to the repression of swarm colony expansion. Interaction between the two cognate proteins reduces swarm restriction (Saak and Gibbs, 2016). IdsD and IdsE proteins may constitute a lethal effector-immunity (toxin–antitoxin) system. Contrary to QS, which is based on contact-independent recognition, T6SS-associated recognition generally requires cell-to-cell contact (Saak and Gibbs, 2016). *P. mirabilis* uses the T6SS to discriminate between strains, coordinate multicellular swarming behavior, and direct its collective movement. Thus, the T6SS is essential for boundary formation and mediates cell–cell communication of swarming *P. mirabilis* via specific self-identity determinants.

### The T6SS of the *P. fluorescens* MFE01 Strain

The *P. fluorescens* MFE01 strain, like numerous other *P. fluorescens* strains, does not produce the QS signals of *P. aeruginosa* (no HSL or PQS) (Gallique et al., 2017). MFE01 is an aggressive T6SS strain which contains a unique T6SS core component cluster and three orphan *hcp* genes (Decoin et al., 2014, 2015). The MFE01 T6SS is involved in biofilm formation and maturation (Gallique et al., 2017), as shown for other T6SSs (de Pace et al., 2011; Sheng et al., 2013; Lin et al., 2015; Tian et al., 2015). Indeed, *P. fluorescens* MFE01 is unable to form biofilms once the T6SS machinery is inactive (in a *tssC* mutant), whereas individual mutations of the three *hcp* genes affect biofilm maturation, but not formation. Intra-bacterial cooperation in conditions of biofilm formation via T6SS dueling could occur. Indeed, alterations of membrane phospholipid composition increase the ppGpp stress-response signal, which in turn causes the premature production of HSL-QS signals, including in *P. aeruginosa* (Baysse et al., 2005) (**Figure 2**). Similarly, communicating pathways could be activated following membrane perturbation due to T6SS perforation in MFE01 strain during “tit-for-tat” interactions.

## Conclusion

A recent study showed that bacteria can reuse T6SS components from attacking cells for new T6SS assembly (Vettiger and Basler, 2016) (**Figure 1**). This suggests that an increase in cell density increases the concentration of T6SS components in bacteria and the ability of the cell to fire again, forming a positive feedback loop. We postulate that T6SS could be a cell-to-cell signal between sibling cells, depending on cell density, similar to the QS pathway, especially in bacteria devoid of QS signals.

## Author Contributions

Writing, review, and editing: MG, AM, MB.

## Conflict of Interest Statement

The authors declare that the research was conducted in the absence of any commercial or financial relationships that could be construed as a potential conflict of interest.
